# Development of a community health inclusion index: an evaluation tool for improving inclusion of people with disabilities in community health initiatives

**DOI:** 10.1186/s12889-015-2381-2

**Published:** 2015-10-13

**Authors:** Yochai Eisenberg, James H. Rimmer, Tapan Mehta, Michael H. Fox

**Affiliations:** Institute on Disability and Human Development, 1640 W. Roosevelt Rd. M/C 626, Chicago, IL 60608 USA; University of Alabama at Birmingham/Lakeshore Foundation Research Collaborative, 4000 Ridgeway Dr., Birmingham, 35209 AL USA; Division of Human Development and Disability, National Center on Birth Defects and Developmental Disabilities, Centers for Disease Control and Prevention, 1600 Clifton Road, MS E88, Atlanta, GA 30333 USA

**Keywords:** Physical activity, Nutrition, Obesity, Disability, Community health, Accessibility, Built environment

## Abstract

**Background:**

Community health initiatives often do not provide enough supports for people with disabilities to fully participate in healthy, active living opportunities. The purpose of this study was to design an instrument that focused on integrating disability-related items into a multi-level survey tool that assessed healthy, active living initiatives.

**Methods:**

The development and testing of the Community Health Inclusion Index (CHII) involved four components: (a) literature review of studies that examined barriers and facilitators to healthy, active living; (b) focus groups with persons with disabilities and professionals living in geographically diverse settings; (c) expert panel to establish a final set of critical items; and (d) field testing the CHII in 164 sites across 15 communities in 5 states to assess the instrument’s reliability.

**Results:**

Results from initial analysis of these data indicated that the CHII has good reliability. Depending on the subscale, Cronbach’s alpha ranged from 0.700 to 0.965. The CHII’s inter-rater agreement showed that 14 of the 15 venues for physical activity or healthy eating throughout a community had strong agreement (0.81 – 1.00), while one venue had substantial agreement (0.61 – 0.80).

**Conclusion:**

The CHII is the first instrument to operationalize community health inclusion into a comprehensive assessment tool that can be used by public health professionals and community coalitions to examine the critical supports needed for improving healthy, active living among people with disabilities.

**Electronic supplementary material:**

The online version of this article (doi:10.1186/s12889-015-2381-2) contains supplementary material, which is available to authorized users.

## Background

Over the past decade, studies have pointed to the importance of addressing built and social environments for promoting healthy behaviors [1, 2]. Existing *Healthy Communities* programs, funded by the U.S. Department of Health and Human Services since 2003, have focused on policy, systems and environmental (PSE) changes to promote active living, healthy eating and weight management. Community coalitions and partnerships work together nationally on implementing PSE improvements as a way to instill healthy living opportunities throughout communities, increasing access to community health resources, and making healthy options the default [3, 4]. PSE strategies intend to impact population health, yet there has been little research examining how PSE strategies impact different sub-populations within communities [5].

People with disabilities comprise 12 – 18 % of the U.S. adult population, or 37.5 – 56.7 million people [6, 7]. This segment of the population constitutes a diverse subset of individuals who experience limitations in mobility (difficulty or inability to walk), cognition (developmental/intellectual disabilities or behavioral/emotional disorders), and/or sensory function (vision/hearing difficulties). As a sub-population, people with disabilities are more likely to be sedentary [8–10], have greater health problems [11–14], and experience more barriers to participating in physical activity and good nutrition compared to the general population [15–17].

Determining changes in PSE have been difficult to measure, but increasingly process evaluations and survey tools are being developed to measure these changes [18, 19]. Of concern, however, is that the instruments currently being used to measure physical activity and healthy eating at the community level are not designed to comprehensively assess the scope and depth of factors that impact people with disabilities, including a growing number of baby boomers who are aging *into* disability [20]. Existing instruments developed to audit PSE for the general population, such as the Community Healthy Living Index (CHLI) [21] and the Community Health Assessment aNd Group Evaluation tool (CHANGE) [22], include some items that apply to environmental accessibility and policies for people with disabilities, but lack the scope and depth necessary to detect potential problems that people with disabilities may encounter when attempting to access these services.

**Fig. 1:**
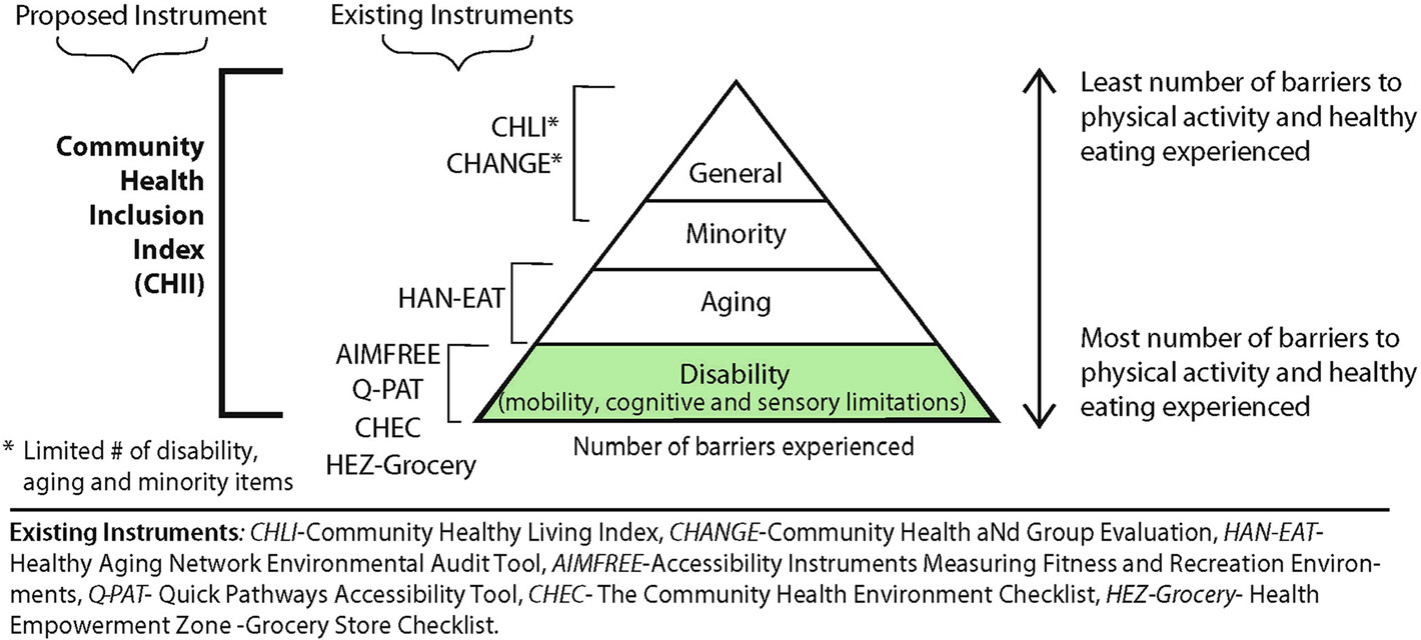
Rationale for the design of the Community Health Inclusion Index (CHII)

Figure 1 presents a visual representation of barriers associated with community healthy living by four specific subgroups; people with disabilities, older adults, minorities and the general population. The greatest number of barriers occurs among people with disabilities who have one or more mobility, cognitive or sensory limitations. These barriers can impose significant restrictions on use of community-based facilities, programs and services, often to a much greater degree than the general population [23]. While there are a few micro-level audit tools (as illustrated in Fig. 1) developed to address issues of accessibility to healthy venues and services affecting people with disabilities, they often target one disability subgroup or a singular area of health promotion. These instruments also focus on targeted points in the community such as fitness centers (AIMFREE), grocery stores (HEZ-Grocery Checklist), building usability (CHEC) or the pedestrian environment (Q-PAT and HAN-EAT). To date, there are no published instruments that provide a comprehensive, global assessment of community health inclusion, a term used to describe the broad range of access to community- level healthy, active living opportunities for people with a range of functional limitations.

The purpose of this study was to develop a community health inclusion measurement tool that would identify key barriers and facilitators to a broad range of community-level issues that affect participation by adults and children with disabilities in healthy living initiatives. The goal was not to replace existing tools that function at the micro-level, but rather, to develop an instrument with a new purpose that would bridge the gap between more targeted, micro-level audits specifically designed for people with disabilities (AIMFREE, CHEC, HEZ-Grocery Checklist, Q-PAT) and community level tools focused on the general population (CHANGE & CHLI). We refer to our instrument as the *C*ommunity *H*ealth *I*nclusion *I*ndex or CHII (pronounced, “CHEE”). This paper describes the development of the CHII and its potential to support greater independence and healthy, active living for people with disabilities in community settings.

## Methods

### Instrument development – content validity

The CHII was developed in three phases: (1) literature review, (2) focus groups, and (3) expert panel review. Each is described below.

#### Literature review

A comprehensive set of items related to barriers and facilitators to healthy, active living for adults and children with disabilities were first identified in existing literature, building on the extensive review of built environment instruments completed by Gray [20]. Instruments were selected that either reported psychometric properties, or were considered highly influential and currently used in public health practice. In addition, items were gathered from published studies that examined barriers and facilitators to healthy living for people with disabilities. Database searches included NARIC, PubMedAHL, and SportsDiscus, and books and grey literature identified from reports and non-peer reviewed magazines. Key search terms were used and articles screened for their relevance to people with disabilities. Among articles that met these criteria, the specific barriers and facilitators described in the manuscripts were extracted for potential use in the CHII.

#### Focus groups

Twenty semi-structured focus groups were conducted in geographically diverse communities across the U.S. from the East Coast, Midwest, Southeast, and Northwest. The focus groups were conducted in a diverse set of communities, with most coming from low and medium income communities. Eight were in rural and 12 in urban communities. The interviews were evenly split among individuals with disabilities (group 1) and professionals (group 2) who work with people with disabilities. The inclusion criteria for people with disabilities was having a physical, sensory, or cognitive disability and being their own guardian (capable of consenting for themselves). They had to be ≥18 years old for adult focus groups, and between 13–18 years for the youth focus group. For service providers, the inclusion criterion was they had to provide services to people with disabilities. All participants had to speak and understand English.

A purposive sampling framework was used to ensure that in each focus group of people with disabilities, there was good representation from various disability types. The professional focus groups included a diversity of public/private and small/large organizations providing services to a range of people with physical, cognitive and sensory disabilities. Both groups were recruited through local partners and their existing networks through email and website postings.

The major theme of the focus groups was a discussion of the facilitators and barriers to community health inclusion structured around five domains: Built Environment, Equipment, Programs/Services, Staff and Policies. These domains had been established in a previous study by Rimmer et al. as being associated with community health inclusion [24]. The focus groups were stratified so that sectors of the community, previously identified by the CDC [22] (schools, health care facilities, workplaces, community institutions/organizations and the community-at-large), were discussed by each of the two respondent groups. Participants were asked about barriers and facilitators they experienced to being physically active or accessing healthy foods within their assigned sector. For instance, young adults with disabilities in high school provided feedback about school-related issues, while working-age adults with disabilities provided feedback about work sites.

Focus group recordings were transcribed verbatim and coded by two trained coders using content analysis into barriers and supports for healthy, active living. Content analysis can be used to organize and distil content in to a few categories that share similar meanings and explain the phenomena under study. Inductive content analysis (used in this paper) involves open coding and development of code sheets of the categories the coders have decided on based on their interpretation of similar meanings [25]. Inter-rater agreement was assessed by dividing the agreed upon codes obtained from the coded transcripts by the total number of codes [26]. Written informed consent for participation in the focus groups was obtained from participants or, where participants were youth, a parent or guardian.

#### Development of the item bank

Items from focus groups, existing instruments, and articles on barriers/facilitators were compiled into a master item bank. Two trained research staff reviewed each item to determine if they met two criteria: 1) could it be objectively measured? and 2) did it relate to physical activity, nutrition or obesity at the PSE and not personal level? Items that did not meet these criteria were excluded. Duplicate items were combined. The research team grouped these items into a set of constructs that measured a common theme, which could be representative of a physical feature (e.g. sidewalks), program (e.g. nutrition class) or a policy (e.g. staff training). For instance, items on sidewalk length, slope, condition, materials, and connectedness were aggregated into a construct on sidewalks.

#### Expert panel

Twenty national experts, identified from the literature and through professional networks, were recruited to review the item bank. One fourth of the experts identified as having a disability. Eligibility criteria required all experts to have a background working with people with disabilities in one or more content areas related to physical activity or nutrition. The experts were organized into four panels based on their area of expertise (physical activity, nutrition, general accessibility and community design). Panels were charged with reducing the number of items to those most critical for measuring community health inclusion. An item was considered critical if it was necessary for people with disabilities to 1) independently access the physical activity or food environment, and 2) be able to participate in health promotion activities such as classes, programs, and services.

Review of items was conducted in two rounds using electronic surveys. Each expert panel had five members who reviewed materials related to their area of expertise. During the first review, experts were asked to decide on importance and level of measurement (narrow focus or broad) of each construct. In the second review, they decided on which items best served as indicators most representative of accessibility for each category. Items with less than two votes, out of a possible score of 5, were removed unless an expert had a strong rationale for keeping it, in which case it went through an additional review by the research team to determine its distinctiveness from other items.

#### Instrument design

**Fig. 2:**
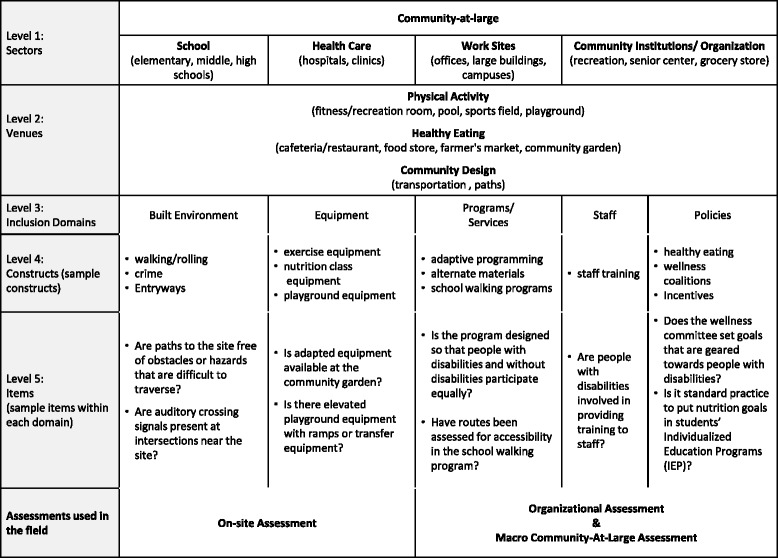
Structure of the CHII

The structure of the instrument is hierarchical in nature (sector-venue-domain) and was designed so that comparisons could be made across the sectors illustrated in Fig. 2. These sectors have been used in other national instruments [21, 22] and represent a common framework for examining a community’s healthy living resources. The CHII was organized by venues within each sector. For example, a cafeteria is considered a venue that is located in three different sectors: hospitals, schools and work sites. Each sector had a few additional items relevant to the specific populations that used the sector (e.g., children in schools). The Community-at-Large sector incorporated items that were about the community as a whole. Within each sector, there are items across five domains: Built Environment, Equipment, Programs/Services, Staff and Policies. The items under the built environment and equipment domains were conducted through an observational audit of the facility, while the items under the program, staff and policy domains required an interview with the management of the organization.

The Survey Research Laboratory (SRL) at the University of Illinois at Chicago (UIC) worked with the project team to develop wording and order of the instrument. The SRL Questionnaire Review Committee, composed of technical experts in survey design, reviewed and approved the instrument for field testing.

#### Cognitive response testing

Pilot testing of the initial instrument was carried out at eight sites from different sectors (3 schools, 1 work site, 1 hospital, 1 grocery store, and 2 park district recreational sites) around the Chicago area. The focus of this testing was to determine how well the order and protocol worked for the observational audit and to obtain feedback from respondents on the interview portion of the survey related to content and clarity of questions. Feedback from respondents was used to modify the interview section of the CHII. Feedback from raters on the observational assessment was used to reorganize items and ensure protocol clarity.

#### Sampling and field testing sites

Academic partners within five states (Illinois, North Carolina, Arizona, Alabama and Montana) were involved in the field testing of the CHII. A purposive sampling methodology, whereby the sample is selected based on knowledge of the population of all communities, was used to select communities with varying levels of income, urbanity and geographic distribution using data from the American Community Survey and the United States Department of Agriculture [27]. Fifteen communities were identified based upon each combination of income and urbanity type. Within each community, raters assessed Schools, Work Sites, Health Care Facilities, Community Institutions/Organizations and the Community-at-Large.

An academic partner in each state coordinated the field testing locally. Raters were recruited who had knowledge of accessibility and inclusion and some experience with conducting assessments. Additionally, partners were asked to ensure that some of the raters were people with disabilities. Most of the raters (60 %) lived in the community that they were rating. Local knowledge of the community proved to be important for recruitment of field testing sites. Some raters (47 %) were recruited from Centers for Independent Living, organizations devoted to providing improved opportunities for people with disabilities within communities [28].

All raters were trained by the project director (YE) during a 2-day workshop in: (1) using the CHII in field-based sites; (2) learning how to conduct formal interviews for collecting data from management of organizations; and (3) practicing the use of two measurement tools related to ADA accessibility items (door pressure gauge and a smart tool to measure slope), which are commonly used in accessibility assessments and based on Americans With Disabilities Act Accessibility Guidelines, or ADAAG [29].

While field testing the CHII, raters were asked to sample three sites from each of the following sectors: schools, work sites, health care facilities, community institutions and food sites using inclusion criteria of: public schools, large employers, hospitals or clinics, community institutions/organizations, and either grocery stores, farmers markets or community gardens. Raters were instructed to identify sites that had physical activity and/or nutrition venues or programs, which were defined in the CHII structure (Fig. 2). Two raters went to each site to obtain inter-rater agreement for each type of venue (Level 2 in Fig. 2). Raters pre-identified transit stops, paths and venues and then proceeded to independently rate each site.

Focus group methods and field testing procedures were approved by the University of Illinois at Chicago’s Institutional Review Board (FWA #00000083) under expedited review. Raters worked with state coordinators to select and recruit sites that met the criteria using approved scripts and recruitment materials. Recruitment occurred by phone, email and in-person, where applicable. Permission was requested for all sites before conducting an assessment. No informed consent was needed for field testing (per IRB) as there were no human subjects; the subject was the site being assessed.

### Reliability analysis

Paper surveys were scanned and coded using electronic data recognition software [30]. Raters in one of the states used PDF copies on tablets to collect data, which was an important adaptation that allowed raters with less upper body motor control to fill out the CHII. Surveys were then quality checked by a graduate assistant before exporting to SPSS [31].

We conducted two types of analyses: reliability analyses by estimating internal consistency and inter-rater agreement (IRA). Internal consistency measures the ability of the items within a construct to arrive at consistent results. Constructs were analyzed across sectors and for particular venues to measure internal consistency. Constructs with Cronbach’s alpha ≥ 0.700 were considered reliable and retained. Constructs that met this criterion after removing one of the items were also retained [32–34]. Cronbach’s alpha was computed using SPSS version 21.

Inter-rater agreement (IRA) is used to assess the extent to which multiple raters assign the same precise value for each item being rated [35]. We choose IRA instead of Inter-rater reliability because of the multi-center study design that utilized multiple pairs of community based raters. IRA was computed at the venue-level using Kendall’s coefficient of concordance W and percent agreement for all items that were part of the observational assessment using R package Inter-rater reliability (IRR) version 0.84. For every venue, Kendall’s W was computed for each pair of raters. The average of all Kendall’s W across all rater pairs was considered as the estimate of the IRA for a venue. No IRA was calculated for the interview portion of the survey as the information came from the respondent and was not an assessment by the raters. We also estimated percent agreement using pairs of rater’s assessments per venue. We used benchmarks of agreement similar to the ones established by Landis and Koch (slight: 0–0.20 slight, fair: 0.21–0.40, moderate: 0.41–0.60, substantial: 0.61–0.80, and almost perfect: 0.81–1) [36].

## Results

**Fig. 3:**
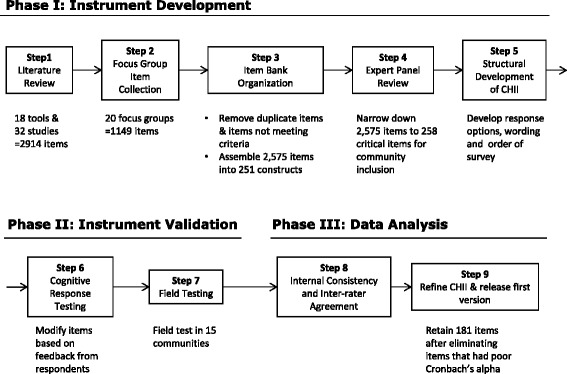
Steps for the development of the CHII

The development of the CHII and the sequential phases used for item refinement, reduction and data analysis are described in Fig. 3. The corresponding sections below describe the number of items identified from each source, the number of items dropped, and some examples of items not included.

### Literature review

Eighteen existing instruments were identified as potential sources of important items for the CHII. Five hundred and thirteen manuscripts were identified from the literature search. Thirty two manuscripts met the study criteria. Manuscripts that did not discuss barriers and facilitators associated with health promotion and disability were excluded from the analysis. Based on the 18 tools and 32 studies identified, 2914 items were identified for possible inclusion in the CHII. See Additional file 1 for details about the tools and studies found in the literature review.

### Coding of focus group items

There were 1149 potential items that were coded as part of the focus group analysis. The percentage of inter-rater agreement on transcribed focus group recordings was 0.97, with a range from 0.94 to 1.00. Minor disagreements had to do with aspects of the built environment and transportation and whether or not a code was meant as a facilitator or barrier. Additional file 2 includes demographic information about the 159 focus group participants as well as examples of the barriers and facilitators coded through the focus groups and organized by CHII domain.

### Item bank refinement

The item bank screening resulted in 1488 items being dropped. Reasons for removal included: having duplicates, not objectively measurable, and describing a personal factor vs. a PSE factor. The final item bank was composed of 2575 items across 5 sectors and 5 domains. Categorization of items resulted in 251 constructs.

### Expert panel

The expert panel reviewed the 251 constructs in the first round and 55 were retained. The types of constructs that were removed because they were viewed as non-essential ranged from saunas/hot tubs, to healthy food purchasing, to weather. In the second round 407 items were reviewed and 149 were removed. Twelve items were included that did not have enough votes but had strong arguments from experts and were considered distinct by the research team. Items removed in the second round included such items as adequacy of the paratransit service area, continuing education for staff to work with people with disabilities, and whether or not programs require doctor’s notes for participation. Experts reduced the number of items to 258, which were included in the final design of the instrument. Per suggestions of experts some constructs were split up to arrive at a total of 66 constructs. Additional file 3 shows the items kept and removed by the expert panel in the second round.

### Cognitive response testing

Responses indicated that 83 % percent of the interview questions were clearly written and did not require any further elaboration. Feedback on the remaining items was used to clarify items prior to field testing. Respondents had some difficulty understanding certain terms, not having a N/A option and differentiating between segregated vs. integrated program activities. Relevant questions were cleared up by providing definitions for key terms such as inclusion, accommodation, and adapted equipment, adding a N/A option, and emphasizing that questions were designed with integrated programming in mind. Language was inserted into the protocol to help remind the respondent that the questions were only going to be used for pilot testing the CHII and not to evaluate the facility.

### Summary description of the field testing

A total of 164 sites were assessed that included 27 schools, 30 work sites, 32 health care facilities, 37 community organizations and 38 food sites. Table 1 summarizes the sample characteristics used in field testing the CHII at the Community, Sector and Venue levels. The most common physical activity venue assessed was fitness/exercise rooms and the most common food venue was cafeterias/restaurants.

**Table 1:** Characteristics of CHII field testing sample (*n* =15 communities,164 sites, 263 venues)

Communities^a^	*n*	Sector types	*n*	Venues	*n*
**Urban/Rural**	**15**	**Work sites**	**30**	**Physical activity Venues**	**156**
Urban	7	Office (i.e. suite)	5	Trails	8
Suburban	5	Building as a whole	22	Sports field	23
Rural	3	Campus	3	Playgrounds	24
**Household Median Income**	**15**	**Schools**	**27**	Pools (indoor & outdoor)	11
Low	6	Elementary	10	Fitness/exercise rooms	57
Medium	7	Middle	7	Gyms	33
High	2	High	10	**Food Venues**	**107**
**% Disability**	**15**	**Health care facilities**	**32**	Grocery stores/food store	27
<10 %	6	Clinics	22	Cafeterias/Restaurants	65
10–15 %	5	Hospitals	10	Farmers markets	6
>15 %	4	**Community institutions**	**75**	Community Gardens	2
		Community Based Org	7	Nutrition programs	7
		Recreation Center	26		
		Senior Center	4		
		Food sites	38		

^a^Community level metrics obtained from the American Community Survey 2008–2012

BOLD - measures of communities as well as categories of sectors and venues

Of the sites initially contacted, 43 % agreed to participate in the study. Food sites and community organizations were the most successfully recruited sectors (69 % and 65 %, respectively), followed by health care (44 %), work sites (33 %) and schools (23 %).

### Reliability testing

Table 2 reports the constructs that met our internal consistency cutoff of 0.700. Of the 66 constructs assessed during field testing, 31 met the threshold of internal consistency. The total number of items (258) was reduced to 181.

**Table 2:** Internal consistency of CHII constructs measured using Cronbach’s Alpha

Constructs	Items	Alpha
Built Environment		
Paths from intersections	9	0.965
Crime	9	0.890
Locker Room	9	0.849
Intersections	6	0.810
Waiting Room Accessibility	4	0.790
Transit Accessibility	12	0.761
Multi-use Trails	5	0.740
Cafeteria Accessibility	9	0.720
Routes to outdoor venue	8	0.720
Routes to indoor venue	7	0.720
Restrooms	6	0.711
Negative walking features	5	0.710
Menus	2	0.710
Parking accessibility	4	0.706
Appealing walking features	7	0.704
Entrances	11	0.702
Exam room accessibility	7	0.700
Equipment		
Exam room equipment	8	0.759
Aisles in fitness areas	2	0.751
Gym	3	0.737
Playground	2	0.703
Program		
School Walking program	3	0.866
Physical activity materials	4	0.771
Healthy food promotion	4	0.731
Physical activity programs	4	0.702
Nutrition Materials	3	0.700
Policy		
Wellness coalition	3	0.941
Healthy Eating Policy	6	0.743
Wellness coalition inclusion	3	0.703
Work site incentives	7	0.700
Staff		
Staff Training	9	0.700

Table 3 reports the Inter-rater agreement (IRA) using Kendall’s W and percent agreement for each of the venues assessed during the field testing. Based on the mean across the pairs of raters, all of the venues showed almost perfect (0.81- 1.00) agreement except for sports field, which showed substantial (0.61 – 0.80) agreement. Certain venues had too small of a sample to calculate IRA. These included pools, farmers markets and community gardens.

**Table 3:** Venue subscale inter-rater agreement (IRA)

		Kendall's W				Percent agreement			
Venue (# of items)	# of rater pairs	Mean	Std. dev	Min	Max	Mean	Std. dev	Min	Max
Physical activity									
Gym (9)	12	**0.95**	0.08	0.80	1.00	**0.92**	0.11	0.67	1.00
Fitness room (8)	13	**0.92**	0.11	0.73	1.00	**0.86**	0.17	0.60	1.00
Playground (10)	10	**0.92**	0.15	0.50	1.00	**0.81**	0.13	0.67	1.00
Trails (13)	6	**0.90**	0.10	0.75	1.00	**0.80**	0.23	0.44	1.00
Sports Field (8)	8	**0.78**	0.21	0.50	1.00	**0.86**	0.15	0.63	1.00
Food									
Cafeteria (15)	15	**0.92**	0.09	0.73	1.00	**0.86**	0.13	0.54	1.00
Grocery (12)	12	**0.89**	0.12	0.66	1.00	**0.81**	0.17	0.50	1.00
Health Care									
Doctor Office/Clinics (27)	14	**0.89**	0.13	0.50	1.00	**0.87**	0.10	0.63	1.00
Community at Large									
Transportation (12)	13	**0.87**	0.10	0.66	0.99	**0.85**	0.11	0.62	0.99
Community Design (30)	15	**0.87**	0.07	0.78	1.00	**0.80**	0.09	0.68	1.00
General Access at Sectors									
Health care (34)	13	**0.92**	0.07	0.77	1.00	**0.90**	0.08	0.79	1.00
Work site (34)	11	**0.92**	0.08	0.78	1.00	**0.82**	0.30	0.00	1.00
School (34)	10	**0.90**	0.09	0.76	1.00	**0.89**	0.11	0.71	1.00
Community Institution/Organization (34)	14	**0.89**	0.08	0.77	1.00	**0.91**	0.06	0.82	1.00
Food Site (25)	14	**0.89**	0.09	0.72	0.99	**0.89**	0.14	0.43	1.00

BOLD – key measure of IRA used

## Discussion

The results of this study support the use of the CHII as a community-based health assessment tool that can be of value in designing policies, systems and environments that represent the physical activity and nutritional needs of adults and children with disabilities. The three primary elements of validation — internal consistency, inter-rater agreement and content validity based on an expert panel and focus groups — demonstrated fair to good psychometric properties.

While there has been a great deal of positive movement in *healthy community* initiatives across the U.S. and Canada, improvement in general health indicators from these efforts may not be generalizable to adults with disabilities. Given the high number of barriers that people with disabilities encounter when accessing community health/wellness services [16, 37, 38], the CHII can provide a better understanding of what some of the concerns are related to accessing existing or new programs or services by community members with disabilities.

The CHII is the first community health inclusion instrument to assess disability inclusion in policy, systems and environments that support healthy living. The instrument stands out from existing micro-level audit tools by focusing on multiple sectors in the community, covering both nutrition and physical activity content areas, and applying a cross-disability approach. The CHII adds the critical missing component of universal design to currently used community level tools (e.g., CHANGE, CHLI).

The CHII was also designed to work in conjunction with existing community level assessment and micro-level audit tools. Communities that are using instruments to evaluate access to health-oriented programs, services, and policies can incorporate the CHII into similar community sectors.

The results of the CHII can be useful at multiple levels. At the Community-at-Large level, it can assist policy makers, public health officials and disability organizations in understanding to what extent residents have access to health promoting sites that are inclusive, which is something that can be monitored over time and in relation to PSE interventions. For organizations completing the CHII, a profile of inclusive and non-inclusive structures, programs, services, etc. can be established across the different domains and constructs, serving as a benchmark for their level of health inclusion and as an aid in developing goals for future implementation. Findings from the CHII can also be communicated locally to help people with disabilities and disability organizations become aware of the variety of inclusive sites within a community. Various channels can be used for dissemination, including independent living centers, local parent groups and through service providers. Local advertising of inclusive sites can promote the use of new healthy, living opportunities.

In the future, we anticipate that the CHII will be used by local health departments and organizations working on PSE initiatives, or among disability organizations and centers for independent living interested in mapping out levels of inclusion in their communities. Organizations can also conduct assessments on their own facilities to better understand their level of inclusion. The CHII can be accessed on-line through the National Center on Health Physical Activity and Disability website (www.nchpad.org), where training on using the CHII has been distilled into an illustrated manual and set of instructional videos.

### Limitations

The electronic surveys used in the expert panel validation process limited interaction among members. If time and funding would have allowed, an in-person expert panel review may have resulted in more personal interactions between members discussing items between each other. While the sample of communities used in the field testing of the CHII was diverse geographically, economically, by urbanity, and size, it cannot be considered a representative sample of U.S. communities as sites were not selected through random assignment. Criterion validity was not assessed as part of this study because there were no valid/reliable assessment tools that measure community health inclusion to serve as the criterion.

Similar to other community assessment tools, gaining access to facilities was a potential limitation in using the CHII. The recruitment of field testing sites for schools and work sites was particularly challenging. Several of the schools that were approached stated they had other priorities and reporting obligations. Some community raters reported that sites perceived they were under investigation or feared repercussions if the survey results reflected poorly on the accessibility of their facility. There is potential that some sites who didn’t agree to participate knew they had very low accessibility and didn’t want to be identified. In some areas, having a credible community contact facilitated the successful recruitment of sites. In other areas, having official municipal agency support also resulted in buy-in from potential sites. These difficulties in recruitment highlight the need for community-level tools to be integrated within broader community coalition efforts where mutual trust, political commitment and partner buy-in have been well established. As recommended when implementing other community assessment tools [3–5, 21, 22], we also suggest that communities desiring to build *inclusive healthy communities* work within a broad, comprehensive community initiative that leads to a useful planning process, which is supported by local and state efforts [39]. Due to the low sample size of venues that were assessed by the same two raters, we were not able to estimate inter-rater reliability (IRR). However, the inter-rater agreement does provide an estimate of the stability of scores within a venue [35]. Finally, item reduction based on Cronbach’s alpha is only one approach. Comparing two approaches to item reduction: a) maximizing Cronbach’s alpha and b) Rasch item-fit analyses Erhart et al. suggested that neither of those two approaches were superior and it may be appropriate to use both of these analyses [33]. In fact, item-reduction using Cronbach’s alpha could be considered a conservative approach (greater item reduction) given that Cronbach’s alpha underestimates reliability severely when the ‘tau-equivalent’ assumption is not met. The ‘tau-equivalent’ assumption is sensitive to lack of underlying unidimensionality and the number of test items [40]. Although our current study, due to the low sample size, did not include Rasch analyses for unidimensionality of subscales and item-reduction, we intend to pursue this as part of our future work.

## Conclusion

The CHII is a multi-level, mixed-methods instrument that examines community inclusion at sites across different sectors of the community focusing on physical activity and healthy eating. At one level, the CHII assesses an organization’s programs, policies and staff training. At another level, the CHII examines the built environment and equipment from walkability and transportation near the site, to fitness equipment and facilities inside the site. The CHII takes between 1–2 h to complete depending on the number and variety of venues available at a site.

Communities that use the CHII can increase their awareness and knowledge of the areas of need in promoting community health inclusion for people with disabilities.

## Additional files

Additional file 1:
**Literature Review Results.pdf : Includes 1) a flow chart showing the number of manuscripts found and kept as well as sample barriers and facilitators used from this source and (2) a list of instruments selected to be used for building CHII item bank.** (PPTX 35 kb) Additional file 2:
**Focus Group Results.pdf: Shows 1) the demographics of the adults and youth with disabilities as well as the professionals who participated in the focus groups and 2) a list of sample codes developed from focus group transcripts organized by domain of the CHII.** (DOCX 21 kb) Additional file 3:
**Expert Panel Results.xlsx: A database of the items kept and removed by the Expert Panel as part of the development of the CHII.** (XLSX 25 kb)

## Abbreviations

ADA: Americans with Disabilities Act
AIMFREE: Accessibility Instruments Measuring Fitness and Recreation Environments
CHANGE: Community Health Assessment aNd Group Evaluation
CHEC: The Community Health Environment Checklist
CHII: Community Health Inclusion Index
CHLI: Community Healthy Living Index
HAN-EAT: Healthy Aging Network Environmental Audit Tool
HEZ-Grocery Checklist: Health Empowerment Zone Grocery Checklist
IRA: Inter-Rater Agreement
IRB: Institutional Review Board
PSE: Policy, Systems and Environment
Q-PAT: Quick Pathways Accessibility Tool
