# Supplementary material for: Development of a community health inclusion index: an evaluation tool for improving inclusion of people with disabilities in community health initiatives
Source: BMC Public Health. 2015 Oct 13;15:1050. doi: 10.1186/s12889-015-2381-2 (PMC4603756; doi:10.1186/s12889-015-2381-2)
Supplement: Additional file 1: — Literature Review Results.pdf : Includes 1) a flow chart showing the number of manuscripts found and kept as well as sample barriers and facilitators used from this source and (2) a list of instruments selected to be used for building CHII item bank. (PPTX 35 kb) [file 12889_2015_2381_MOESM1_ESM.pptx]

## Slide 1
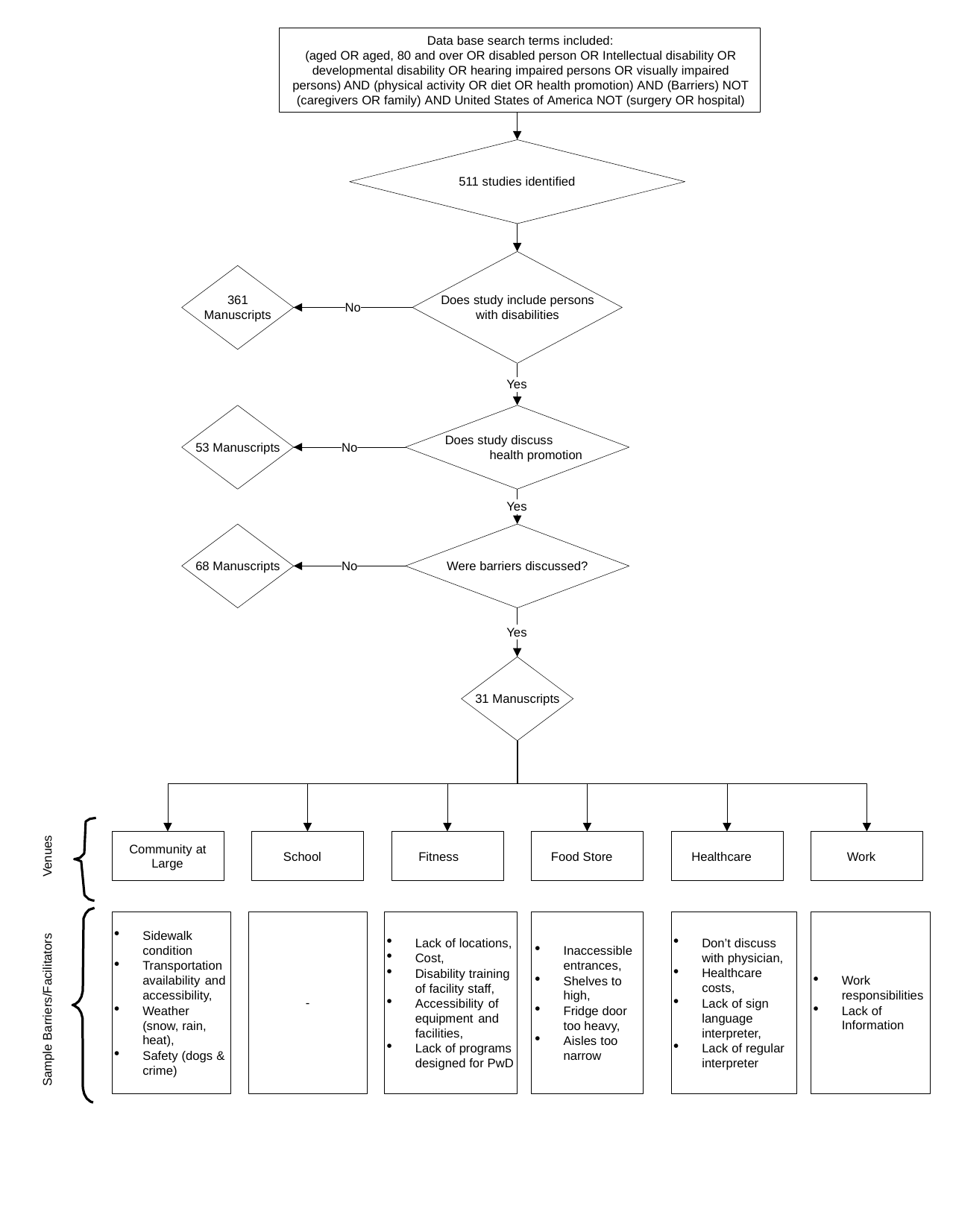

Data base search terms included:
(aged OR aged, 80 and over OR disabled person OR Intellectual disability OR developmental disability OR hearing impaired persons OR visually impaired persons) AND (physical activity OR diet OR health promotion) AND (Barriers) NOT (caregivers OR family) AND United States of America NOT (surgery OR hospital)
511 studies identified
361
Manuscripts
Does study include persons with disabilities
No
Yes
Does study discuss health promotion
53 Manuscripts
No
No
Yes
68 Manuscripts
No
Were barriers discussed?
Yes
31 Manuscripts
Community at Large
School
Fitness
Food Store
Healthcare
Work
Venues
Sidewalk condition
Transportation availability and accessibility,
Weather (snow, rain, heat),
Safety (dogs & crime)
-
Lack of locations,
Cost,
Disability training of facility staff,
Accessibility of equipment and facilities,
Lack of programs designed for PwD
Inaccessible entrances,
Shelves to high,
Fridge door too heavy,
Aisles too narrow
Don’t discuss with physician,
Healthcare costs,
Lack of sign language interpreter,
Lack of regular interpreter
Work responsibilities
Lack of Information
Sample Barriers/Facilitators

## Slide 2
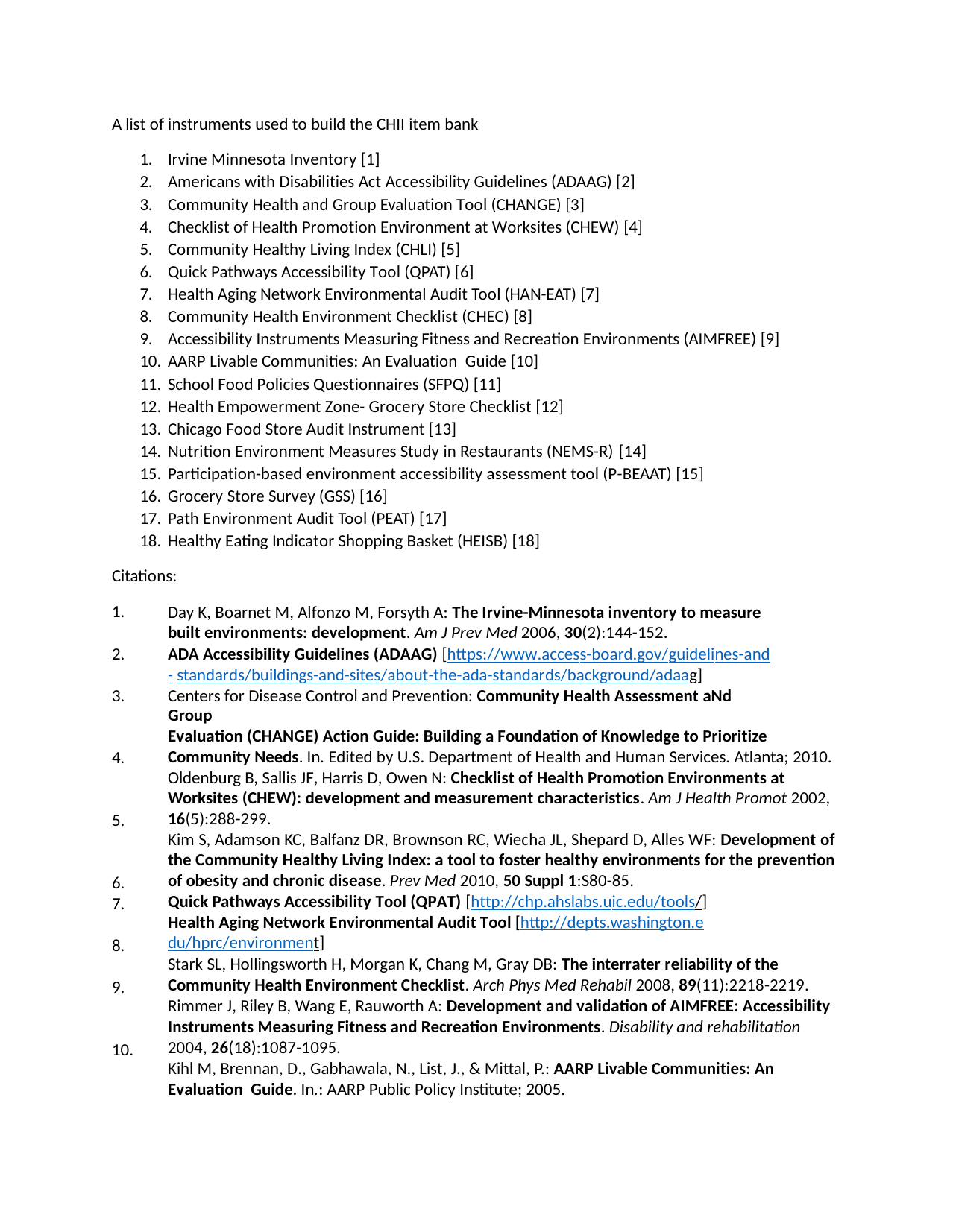

A list of instruments used to build the CHII item bank
Irvine Minnesota Inventory [1]
Americans with Disabilities Act Accessibility Guidelines (ADAAG) [2]
Community Health and Group Evaluation Tool (CHANGE) [3]
Checklist of Health Promotion Environment at Worksites (CHEW) [4]
Community Healthy Living Index (CHLI) [5]
Quick Pathways Accessibility Tool (QPAT) [6]
Health Aging Network Environmental Audit Tool (HAN-EAT) [7]
Community Health Environment Checklist (CHEC) [8]
Accessibility Instruments Measuring Fitness and Recreation Environments (AIMFREE) [9]
AARP Livable Communities: An Evaluation Guide [10]
School Food Policies Questionnaires (SFPQ) [11]
Health Empowerment Zone- Grocery Store Checklist [12]
Chicago Food Store Audit Instrument [13]
Nutrition Environment Measures Study in Restaurants (NEMS-R) [14]
Participation-based environment accessibility assessment tool (P-BEAAT) [15]
Grocery Store Survey (GSS) [16]
Path Environment Audit Tool (PEAT) [17]
Healthy Eating Indicator Shopping Basket (HEISB) [18]
Citations:
1.
Day K, Boarnet M, Alfonzo M, Forsyth A: The Irvine-Minnesota inventory to measure built environments: development. Am J Prev Med 2006, 30(2):144-152.
ADA Accessibility Guidelines (ADAAG) [https://www.access-board.gov/guidelines-and- standards/buildings-and-sites/about-the-ada-standards/background/adaag]
Centers for Disease Control and Prevention: Community Health Assessment aNd Group
Evaluation (CHANGE) Action Guide: Building a Foundation of Knowledge to Prioritize Community Needs. In. Edited by U.S. Department of Health and Human Services. Atlanta; 2010. Oldenburg B, Sallis JF, Harris D, Owen N: Checklist of Health Promotion Environments at Worksites (CHEW): development and measurement characteristics. Am J Health Promot 2002, 16(5):288-299.
Kim S, Adamson KC, Balfanz DR, Brownson RC, Wiecha JL, Shepard D, Alles WF: Development of the Community Healthy Living Index: a tool to foster healthy environments for the prevention of obesity and chronic disease. Prev Med 2010, 50 Suppl 1:S80-85.
Quick Pathways Accessibility Tool (QPAT) [http://chp.ahslabs.uic.edu/tools/] Health Aging Network Environmental Audit Tool [http://depts.washington.edu/hprc/environment]
Stark SL, Hollingsworth H, Morgan K, Chang M, Gray DB: The interrater reliability of the Community Health Environment Checklist. Arch Phys Med Rehabil 2008, 89(11):2218-2219. Rimmer J, Riley B, Wang E, Rauworth A: Development and validation of AIMFREE: Accessibility Instruments Measuring Fitness and Recreation Environments. Disability and rehabilitation 2004, 26(18):1087-1095.
Kihl M, Brennan, D., Gabhawala, N., List, J., & Mittal, P.: AARP Livable Communities: An Evaluation Guide. In.: AARP Public Policy Institute; 2005.
2.
3.
4.
5.
6.
7.
8.
9.
10.

## Slide 3
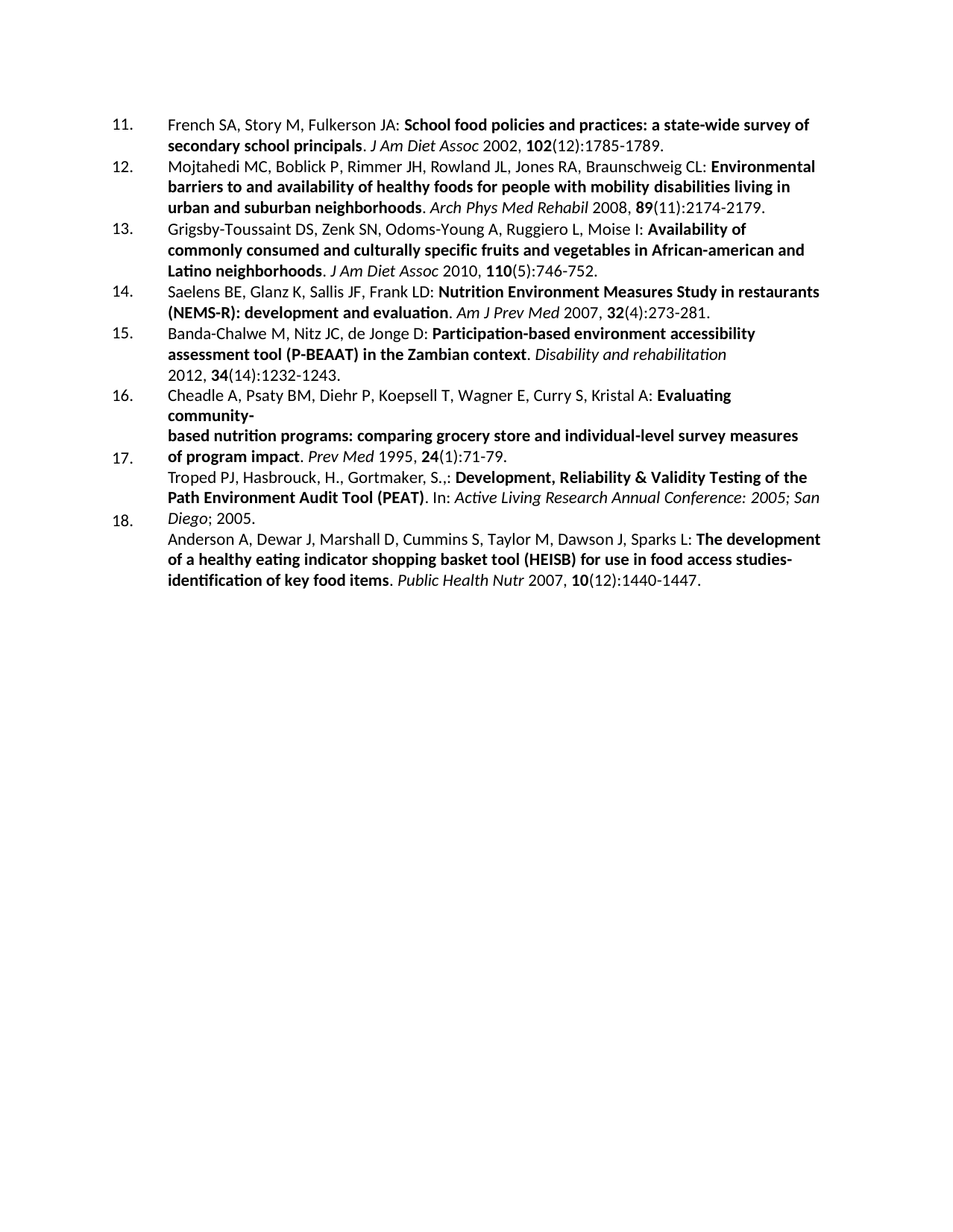

11.
French SA, Story M, Fulkerson JA: School food policies and practices: a state-wide survey of secondary school principals. J Am Diet Assoc 2002, 102(12):1785-1789.
Mojtahedi MC, Boblick P, Rimmer JH, Rowland JL, Jones RA, Braunschweig CL: Environmental barriers to and availability of healthy foods for people with mobility disabilities living in urban and suburban neighborhoods. Arch Phys Med Rehabil 2008, 89(11):2174-2179.
Grigsby-Toussaint DS, Zenk SN, Odoms-Young A, Ruggiero L, Moise I: Availability of commonly consumed and culturally specific fruits and vegetables in African-american and Latino neighborhoods. J Am Diet Assoc 2010, 110(5):746-752.
Saelens BE, Glanz K, Sallis JF, Frank LD: Nutrition Environment Measures Study in restaurants (NEMS-R): development and evaluation. Am J Prev Med 2007, 32(4):273-281.
Banda-Chalwe M, Nitz JC, de Jonge D: Participation-based environment accessibility assessment tool (P-BEAAT) in the Zambian context. Disability and rehabilitation 2012, 34(14):1232-1243.
Cheadle A, Psaty BM, Diehr P, Koepsell T, Wagner E, Curry S, Kristal A: Evaluating community-
based nutrition programs: comparing grocery store and individual-level survey measures of program impact. Prev Med 1995, 24(1):71-79.
Troped PJ, Hasbrouck, H., Gortmaker, S.,: Development, Reliability & Validity Testing of the Path Environment Audit Tool (PEAT). In: Active Living Research Annual Conference: 2005; San Diego; 2005.
Anderson A, Dewar J, Marshall D, Cummins S, Taylor M, Dawson J, Sparks L: The development of a healthy eating indicator shopping basket tool (HEISB) for use in food access studies- identification of key food items. Public Health Nutr 2007, 10(12):1440-1447.
12.
13.
14.
15.
16.
17.
18.
