# Supplementary material for: Development of a community health inclusion index: an evaluation tool for improving inclusion of people with disabilities in community health initiatives
Source: BMC Public Health. 2015 Oct 13;15:1050. doi: 10.1186/s12889-015-2381-2 (PMC4603756; doi:10.1186/s12889-015-2381-2)
Supplement: Additional file 2: — Focus Group Results.pdf: Shows 1) the demographics of the adults and youth with disabilities as well as the professionals who participated in the focus groups and 2) a list of sample codes developed from focus group transcripts organized by domain of the CHII. (DOCX 21 kb) [file 12889_2015_2381_MOESM2_ESM.docx]

| Professionals |  | |
| --- | --- | --- |
| Total sample (n=75) | **n** | **%** |
| **Gender** |  |  |
| Female | 58 | 77 |
| Male | 17 | 23 |
| **Community** |  | 0 |
| Urban | 38 | 51 |
| Suburban | 18 | 24 |
| Rural | 18 | 24 |
| **Population Served** |  | 0 |
| Youth with Disability | 36 | 48 |
| Adult/Physical Disability | 25 | 33 |
| Adult/Sensory Disability | 14 | 19 |
| Adult/Cognitive Disability | 17 | 23 |
| Seniors | 17 | 23 |
| Other | 29 | 39 |
| **Age (years)** |  | 0 |
| 18-44 | 36 | 48 |
| 45-64 | 36 | 48 |
| 65 + | 3 | 4 |
|  |  |  |

| Adults with Disabilities | | | |
| --- | --- | --- | --- |
| Total sample (n=70) | | **n** | **%** |
| **Gender** | |  |  |
| Female | | 43 | 61 |
| Male | | 26 | 37 |
| **Age (years)** | |  |  |
| 18-29 | | 12 | 17 |
| 30-49 | | 28 | 40 |
| 50-64 | | 20 | 29 |
| 65 + | | 10 | 14 |
| **Community** | |  |  |
| Urban | | 35 | 50 |
| Rural | | 33 | 47 |
| **Type of Disability** | |  |  |
| Physical Disability | | 38 | 54 |
| Sensory Disability | | 28 | 40 |
| Cognitive Disability | | 17 | 24 |
| **Assistive Aid** | |  |  |
| Cane | | 13 | 19 |
| Walker | | 13 | 19 |
| Wheel Chair | | 25 | 36 |
| White Cane | | 6 | 9 |
| Animal | | 2 | 3 |
| Hearing Aid | | 5 | 7 |
| **Health** | |  |  |
| Is Physically Active | | 47 | 67 |
| Eats Healthy Foods | | 50 | 71 |
|  | |  |  |
| Youth with Disabilities | | | |
| Total sample (n=14) | **n** | | **%** |
| **Gender** |  | |  |
| Female | 5 | | 36 |
| Male | 9 | | 64 |
| **Age (years)** |  | |  |
| 14 | 2 | | 14 |
| 15 | 5 | | 36 |
| 16 | 3 | | 21 |
| 17 | 4 | | 29 |
| **Community** |  | |  |
| Urban | 9 | | 64 |
| Rural | 5 | | 36 |
| **Type of Disability** |  | |  |
| Physical Disability | 11 | | 79 |
| Sensory Disability | 2 | | 14 |
| Cognitive Disability | 4 | | 29 |
| **Assistive Aid** |  | |  |
| Cane | 0 | | 0 |
| Walker | 3 | | 21 |
| Wheel Chair | 6 | | 43 |
| White Cane | 0 | | 0 |
| Animal | 1 | | 7 |
| Hearing Aid | 0 | | 0 |
| **Health** |  | |  |
| Is Physically Active | 11 | | 79 |
| Eats Healthy Foods | 11 | | 79 |
|  |  | |  |

Examples of barriers and facilitators coded through the focus group analysis organized by CHII domain

| Built Environment Domain Sample Codes | |
| --- | --- |
| **Barriers** | **Facilitators** |
| - Spacing of benches in the locker room does not allow to one to maneuver | - There is adequate outdoor lighting at night |
| - Lack of tactile signs in building | - School cafeteria is accessible for people with disabilities, everything is spaced out |
| - Salad bar is too high to reach from a seated position | - Availability of automatic doors |
| - Each door in a series of doors has a separate door button | - Business provides tables that have different heights (some high, some low) |
| - Automatic lights are on a timer and turn off too soon | - Not Crossing a lot of roads help to make it easier for people with disabilities to travel to venue |
| - Paratransit may not go to the destination (grocery store, fitness center, etc.) | - Automatic doors slide open rather than swing toward people with disabilities |
| - The fitness center walking/running track is too narrow | - Farmers market held on parking lot or street provide level, hard surface for shoppers |
| - Doors close too fast | - The locker room is open and spacious (easy to maneuver in) |
| - Drivers do not yield to pedestrians who are attempting to cross the street | - Accessible ground materials (rubber, chalk) are used in the playground |
| - When sidewalk is cleared of snow, the excess snow is pushed into curb cut which results in a blocked curb cut | - Have ADA accessible water fountains in city parks |
| - Restaurants place straws in inaccessible location | - The restaurant menu is available in Braille |
| - Curb cuts are located only on one side of the street | - There are handicap bathrooms available |
| - The bus stops are far away from destination | - The addition of a crosswalk countdown would help pedestrians cross the street |
|  | - Multiple transportation options are available |

| Equipment Domain Sample Codes | |
| --- | --- |
| **Barriers** | **Facilitators** |
| - There are no individual showers - only one large group shower space - no privacy | - Access to a weight scale that can be used by an individual using a wheelchair |
| - Chairs built into table are too far from cafeteria table - not accessible | - There is open space between exercise equipment |
| - People with disabilities cannot get on exercise bike (no recumbent available) | - The equipment has touch screens (e.g., fitness equipment, restaurants or businesses) |
| - School bus is not accessible for students with disabilities, limits their participation in sports | - Exam tables raise and lower |
| - The wheelchair lift is broken | - The exercise equipment has removable seats so that wheelchairs can get under the equipment |
| - Fitness equipment either located too high or low (e.g. free weights racks) | - Velcro grips help with exercise and physical activity |
| - Mammography equipment will not rotate | - There could be a shopping cart that can be velcroed to a wheelchair or height modified to be lower |
| - The school desks provided are too small |  |

| Programs Domain Sample Codes | |
| --- | --- |
| **Barriers** | **Facilitators** |
| - Wellness program is designed by volunteers who do not have enough time to make program accommodations | - Program is consistently evaluated to determine what is and what is not working |
| - Nutrition signs are not provided in braille or other formats | - Farmers market has a shopping aid available to help the people with disabilities navigate the market |
| - There is minimal or no advertising for the program | - Programs designed to be performed while seated |
| - Families do not know the availability of resources available for students with a disability | - Family of person with a disability allowed to participate in program |
| - Required to have insurance to access program otherwise it is too expensive | - Programs focus on meal preparation and nutritional value |
| - Marketing not used properly to target population (e.g. using computers for elderly, no braille) | - The programs are offered in convenient locations (e.g. people with disabilities can find transportation to them and a bus stop is nearby) |
| - Lack of integration between people with disabilities and people without disabilities in wellness programs | - Program tries to include everyone regardless of disability |
| - The program is available for certain types of disability and not others | - The class/program announcements are available in different forms (e.g. online, print, etc.) |

| Staff Domain Sample Codes | |
| --- | --- |
| **Barriers** | **Facilitators** |
| - Sport coaches do not make accommodations for students with disabilities to participate in sports team | - Teachers demonstrate being physically active |
| - Physical Education teacher is expected to teach adapted physical education but does not know how | - Staff are trained on how to approach people with disabilities and be respectful |
| - Teachers use unhealthy food as reward for doing well in class | - Staff asks people with disabilities how they prefer information at health care site |
| - Doctors speak too fast | - Staff in the fitness facility are available to help the people with disabilities adjust the exercise equipment (e.g. remove the seats) |
| - Staff/Volunteers who led wellness programs only interested in their own personal interest areas | - Staff are able to locate and retrieve healthy food in the business for the people with disabilities |
| - Staff are unsure if what they are doing is rude and lacks etiquette | - People with disabilities are included in the staff training |
| - The parents of adult people with disabilities feel unsafe leaving their child with driver | - Staff teach students with disabilities how to use exercise equipment (to get on/off, adjust etc.) |
| - The staff talks to other people with the people with disabilities not the people with disabilities themselves | - Teachers include people with disabilities |

| Policy Domain Sample Codes | |
| --- | --- |
| **Barriers** | **Facilitators** |
| - Public buses do not run or have very limited time on weekends | - School will make adaptations for student to be able to eat/drink healthy options |
| - School does not provide transportation to sporting venue, all students are required to find their own ride | - The fitness facility is unlocked before and after school to provide access to school gym |
| - Food stamps are not currently accepted at farmers market | - New employees receive disability training during hiring orientation |
| - Cheap food has more unhealthy qualities (e.g. high sodium, and calories) | - Implement disability into company mission statement |
| - Cost of paratransit is high | - Able to take paid time to participate in wellness |
| - Complying with the ADA rules for parks, recreation, and community facilities is expensive | - Only partner with off-site vendors if they are accessible |
| - The administration of a fitness facility does not support buying certain types of equipment, which results in the facility not being able to accommodate people with certain types of disability | - School cannot withhold physical activity as a punishment |
| - The organization is afraid of liability issues and will not allow the people with disabilities to do physical activity or do healthy eating | - Schools replaced unhealthy options with healthy ones |
| - Building policies encourage more spread out buildings making people with disabilities travel farther | - Local government makes sure the building is accessible |
| - Health and wellness not written into students IEP |  |
